# The effect of living alone on the mental health of the economically active floating population during the COVID-19 pandemic

**DOI:** 10.3389/fpubh.2022.931425

**Published:** 2022-08-11

**Authors:** Junzhou Xu, Ling Zhang

**Affiliations:** ^1^Department of Economics and Management, Hubei Polytechnic University, Huangshi, China; ^2^Seoul School of Integrated Sciences and Technologies, Seoul, South Korea; ^3^School of Software and Internet of Things Engineering, Jiangxi University of Finance and Economics, Nanchang, China

**Keywords:** COVID-19 pandemic, living alone, mental health, EAFP, GHQ-12

## Abstract

**Background:**

The Coronavirus disease 2019 (COVID-19) pandemic broke out at the end of 2019 in China. Through a strict Zero-Tolerant strategy, the pandemic was nearly controlled in the first half of 2020, and production resumed in most regions of China. A survey was performed to explore the effect of living alone on the mental health of the economically active floating population (EAFP) in developed regions of China during the COVID-19 pandemic.

**Methods:**

The online cross-sectional survey was conducted in work resumed time in the first half of 2020 in several developed regions of China. The 12-item General Health Questionnaire (GHQ-12) is used to assess the mental health status. The Multi-level ordinary least squares regression was performed on a total of 4,405 samples to examine the relationships between living alone and the participants' mental health.

**Results:**

Many participants lived alone during the COVID 19 pandemic. Living alone is negatively associated with mental health (*p* < 0.01) for EAFP. The effect of living alone on mental health is stronger for females than males and for people with a lover than those without a lover. It is also stronger for the seniors (aged 56–70) than younger ones (aged 16–35), and has no significant influence on the middle-aged population (36–55). The effect is significant for self-employed people and employees, and is not significant for unemployed ones. Furthermore, the right amount of online entertainment can lower the effect of living alone on mental health.

**Conclusion:**

The results show that living alone strongly affected the mental health of EAFP during the COVID 19 pandemic. Moreover, this effect has generated new inequalities among different groups. In addition, to provide more public services to support people against the pandemic, the government should provide more psychological support to those who live alone and guide them to establish a correct view of marriage and love to reduce living alone negative effect and prevent them from mental health problems.

## Introduction

The COVID-19 pandemic broke out at the end of 2019 in China. To contain the outbreak of the pandemic, the Chinese government has taken strict pandemic control rules such as lockdowns and quarantine to achieve the “zero tolerance” target. These strategies have effectively kept the infection rate at a low level in comparison to some other counties. However, the COVID-19 pandemic and its accordingly rigid control rules still affect China's economy. China's Gross Domestic Product (GDP) shrank 1.6% in the first half of 2020, the first contraction since 1976, when the Cultural Revolution ended^1^.

People faced a series of employment problems, such as job loss, income decreased and working time increased, etc., during the COVID-19 pandemic. They have to change their social and work manners, and the usual social and emotional support sources may be cut off. These problems make them increasingly anxious and depressed (1, 2). Industrialization and rapid economic growth have improved China's social conditions and changed people's living arrangements at the same time. Changing lifestyles, reduced family size, and large migration internal and international have led to an increasing number of people living alone, which may even increase to 162 million in 2050 (3).

Living alone is considered worrisome to people's health, because it is widely recognized that humans are naturally other-oriented and enjoy connecting with others (3). Living alone is also linked with anger, social exclusion, and feeling of isolation or dolefulness (4–6), all of which may result in poorer mental health. Furthermore, levels of loneliness are tied up with high level of mindfulness (6, 7). A significant association exists between mindfulness and connectedness to nature (8). When living alone, people may be not mindful, and they are prone to become lonely (9). Therefore, living alone has the potential for depression and anxiety symptoms (10). Living alone is also responsible for morbidity and mortality (11, 12). For those living alone, they are at higher risk of mental health problems during the COVID-19.

Many studies have reported that living alone has a potential association with the mental health (13). Some authors claim that living alone is linked with lower levels of depression (14, 15), and more research findings report that living alone has a positive association with poorer mental health (16–20). Moreover, the findings of (11) report that aside from mental health, the typology of the living alone can be identified by data-driven techniques, differentiated by the number of severity of issues they experienced. Some other works gave the proposals to enhance life quality of living alone people (21, 22).

Little has been done to evaluate the effects of living alone on people's mental health during COVID-19 pandemic. Research (23) explores the effects of living alone on mental health for young adults during the lockdown in India. The results show that young adults living alone are more likely to have mental health problems during COVID-19 pandemic. Another research, aimed at the healthcare professionals' mental health during the COVID-19 outbreak in Spain, reports that more than 80% of participants experience a certain mental health problems, and living alone resulting in loneliness is positively related to higher mental health problems.

Some research has been done to evaluate the moderators of living alone effect on mental health. Gender is a moderator of living alone effect on mental health, and living alone's effect on poorer mental health is more significant for females than males (1, 2). The living alone effect on mental health is also moderated by age, such as the paper of (10) reported that younger individuals who are living alone are prone to have mental health problems. *Hukou* (a kind of household registration in China) is also a moderator between living alone effect and mental health. Residents with Urban Hukou reported more mental health problems related to the COVID-19 outbreak than rural residents (24). Moreover, the factors of social disconnection, subjective loneliness, financial situation, general comfort, preference for living alone, kinds of recreation, relationship quality, and social quality of the community all have moderate effects on the association between living alone and mental health (21, 23, 25, 26).

Previous research mainly examines effects of living alone on mental health and the moderating effect on the link between them. Little has been done in China, study (22) is an exception. It explores the psychological health implications of living alone among middle-aged and seniors in China. However, as far as we know, there is still no research examining the correlation between living alone and mental health for EAFP in the work resumption time during the COVID-19 pandemic. Although the struggle against the epidemic has achieved some success in China, it still depresses China's economy, bringing about a large number of people with reduced income or even unemployment, followed by the loss of financial resources, which have negative impact on their mental health. A number of people lived alone for different reasons during COVID-19 pandemic whether they developed more serious mental health problems or not. More research needs to be done to examine the mental health of those living alone during COVID-19 pandemic to provide evidence to develop measures to improve their mental health. Therefore, it is meaningful to explore the effects of living alone on mental health for EAFP during COVID-19 pandemic.

In this study, the relationship between living alone and mental health has been examined for EAFP in several developed regions of China during COVID-19 pandemic. EAFP refers to a transient population, namely, employees, self-employed ones and unemployed people but seeking a job, doesn't contain economically inactivity ones such as the children, students or retirees. To be specific, the main hypotheses we want to validate in this study are listed as follows.

H1. Living alone has a negative effect on participants' mental health; people living alone are more prone to have mental health problems.

H2. Sex moderates the effect of living alone on participants' mental health. Living alone has a stronger negative effect on the mental health of women than that of men.

H3. Age moderates the effect of living alone on the mental health. Living alone has a more adverse impact on the mental health of senior population compared with the younger population.

H4. Employment status moderates the effects of living alone on the mental health. Living alone has strongest adverse effect on unemployed population's mental health, then self-employed ones, and minimal impact for the employees.

H5. Having a lover moderates the effect of living alone on the mental health. And it is a moderate factor to help enhance mental health of people living alone.

H6. Entertainment on We-Media moderates the effects of living alone on mental health. Right amount of online entertainment can lower the effects of living alone on mental health, and improve the mental health of the living alone EAFP during the pandemic.

## Methods

### Participants and procedure

A cross-sectional research was performed in the regions of the Yangtze River delta region, Guangdong Province and Hunan Province from August 1 to September 30, 2020. The region of the Yangtze River delta region contains Jiangsu Province, Zhejiang Province and Shanghai—some most developed areas in China. They are prosperous and wealthy with their GDP among the top in China's GDP rankings.

An online questionnaire was distributed to the EAFP living in the areas mentioned above. The selection of the participants was based on the following criteria: (a) the respondents must be floating people; (b) they must be people aged 16 or above; (c) They were employed, self-employed or unemployed but seeking a job. Scholars collected the questionnaires from the South China Normal University *via* WeChat (a popular instant message APP in China), and the participants were also encouraged to forward it to others. The survey is anonymous and voluntary. Finally, there were about 4,705 questionnaires completed online. After deleting unfinished responses, 4,405 valid questionnaires were obtained (with a valid response rate of 93.62%).

Of all participants, 1,582 (35.91%) people lived alone during the survey period, and there were 2,158 (48.99%) males and 2,247 (51.01%) females. Most of them (56.35%) thought they were healthy. 60.98% of them have rural hukou, and 2,031 (46.11%) people had completed a college education or above. 1,676 (36.84%) employed participants reported they once lost their job because of the pandemic. Furthermore, more than half of them (2,463, 55.83%) had no kid, 851(19.32%) had one kid, and 880 (19.98%) had two kids. The minimum and maximum mental health score (MHS) of all 4,405 samples are 0 and 12, respectively. The mean of the MHS is 2.285, and the corresponding standard deviation (S.D.) is 2.117.

### Measurements

#### Living alone

As presented in section Introduction, people living alone have higher risks of mental health problems, especially during the COVID-19 pandemic. Here living alone denotes a person who lives with no other people, and only herself/himself lived in a household during the research. Living alone status was collected by asking, “how many people do you live together with now?” Responses ranged from 0 to 6, where 0 denotes the participant lived alone. Living alone is not being single or the official marital status of single, divorced, and widowed. These people may live alone or with others, such as a spouse, parents, or other unrelated persons (27). Living alone is a living arrangement where a person lives with no other people. The living arrangement is not fixed for a person and changes many times during the life course (28). Therefore, living alone is defined here as one person living in a household at the time of the research.

#### Mental health

The dependent variable of the study is the mental health of the participants. The 12-item General Health Questionnaire (GHQ-12) measured the participants' mental health. Since its development by Goldberg in the 1970's, it has been extensively used in different settings and different cultures (29). It is a 12-item rated on a 4-point Likert scale *rarely, occasionally, often*, and *almost always*. The widely used “0-0-1-1” scoring method sums all 12 items scores into a score scale ranging from 0 to 12. A higher GHQ-12 score presents poorer mental health. To test the reliability, Cronbach's alpha coefficient was used to evaluate the internal consistency of the questionnaire, and an alpha ≥0.7 was considered satisfactory (30). The alpha value of GHQ-12 of the entire sample was 0.79, indicating satisfactory results.

#### Personal information factors

For personal information factors, the data collected focuses on sex, age, *hukou*, education, with a lover, the number of children, and employment. Education levels are classified into Junior high school or below, senior high school, and college/university degrees or above. Here with a lover represents a participant who married or has a boyfriend/girlfriend, whether they lived together or not. A lover is his/her soul mate, who can share happiness and woe with him/her. Therefore, with a lover or not may have an effect on the mental health score. Raise children may increase the financial and life burden for a family. Therefore, the number of children is also considered in this part. The current economic activity status; employment is collected for the main work information factor. It is grouped into three parts; employed employees, self-employed workers, and unemployed individuals seeking a job.

#### Lifestyle factors

Individual lifestyle may be associated with mental health. The lifestyles here contain staying up later, no exercise, skipping meals, and entertaining on We-Media platforms (EWP). For EWP, three popular We-Media platforms are considered: DOUYIN, Xinlang Micro-blog, and WeChat public accounts^2^. It was collected by asking three questions; “do you watch short videos through DOUYIN, do you browse on Xinlang Micro-blog, and do you browse on WeChat public accounts?”. The responses are set as very little, occasionally, often, and always. The “0-0-1-1” scoring is used to express the using frequency. The sum of scores for three questions was used to estimate the EWP using frequency.

### Statistical analyses

Description of key variables is analyzed first. Then the ordinary least squares (OLS) regression models are performed to explore the living alone effect on mental health. The heterogeneity of the effect of living alone on mental health among different population groups is examined at last.

## Model specification and data descriptive statistics

To inspect the correlation between *living alone* and the participants' *mental health*, an empirical model is given in *Equation* 1. The explained variable is the GHQ-12 score that reflects the individual mental health denoted as *MHS*. The key explanatory variable is *living alone*, denoted as *VLA*. Other variables about *personal information* and *lifestyle* are control variables. See Equation 1, *i* in the subscript location refers to the *i*th individual in the sample. *VIM* denotes the variable for personal information, and *VLS* is the variable used to represent lifestyles. ε is a random disturbance term.


(1) $$\begin{array}{l} {\text{MHS}}_{\text{i}}=\alpha +\beta {\text{VLA}}_{\text{i}}+\text{γ}{\text{VIM}}_{\text{i}}+\delta {\text{VLS}}_{\text{i}}+{\text{ε}}_{\text{i}} \end{array}$$

## Results

In this section, the descriptive results of GHQ-12 scores under different categories are given first. Then the regression results that reflect the correlation between living alone and mental health are presented, and other factors impacting the participants' mental health are also discussed in the section. At last, the heterogeneity effects of living alone on mental health scores in different groups are explained.

### Descriptive results of GHQ-12 scores under different categories

The descriptive results of GHQ-12 scores under different personal information categories are given in Table 1. The means and the standard deviations (Std. Dev.) are provided in the table. The Mean differences, including Mean differences (Mean-diff) and the *p*-values (*P-*value), are also listed in the table. The first group is the reference group for each variable, and the mean difference is 0. Mean differences of the other groups increase or decrease relative to the standard of the reference group. Take the variable *living alone* as an example; the reference group is the first group (*Live with others*), and the mean difference is 0. Then the mean difference of the second group (*Live alone*) is −0.413, indicating the mean of the group increased by 0.413 relative to the reference group, and this is significant (*p* < 0.001).

**Table 1:** The GHQ-12 mental health scores for each personal information and lifestyle factor.

**Factors**	**Sample size**	**Mean**	**Std. dev**.	**Mean differences**	
				**Mean-diff**	***P*-value**
**Total**	4,405	2.285	2.117		
**Living alone**					
*Live with others*	28,23	2.137	2.074		
*Live alone*	**1,582**	2.550	2.168	−0.413	<0.001
**Sex**					
*Male*	2,158	2.385	2.164	0	
*Female*	2,247	2.190	2.068	0.196	0.002
**Age**					
*16–35*	3,081	2.450	2.148	0	
*36–45*	754	2.036	2.033	0.414	<0.001
*46–70*	570	1.725	1.928	0.725	<0.001
***Hukou***					
*Rural hukou*	2,686	2.264	2.109	0	
*Urban hukou*	1,719	2.318	2.130	−0.054	0.416
**Educational level**					
*J.H. or below*	1,050	2.154	2.124	0	
*Senior high school*	1,324	2.208	2.097	−0.053	0.541
*C/U or above*	2,031	2.403	2.121	−0.249	0.002
***With a lover***					
*No*	1,446	2.551	2.196	0	
*Yes*	2,959	2.155	2.066	0.396	<0.001
**Number of children**					
*0 kid*	2,463	2.525	2.174	0	
*1 kid*	851	2.153	1.999	0.373	<0.001
*2 kids*	880	1.880	2.005	0.646	<0.001
*3 kids or above*	211	1.706	1.944	0.819	<0.001
**Employment**					
*Employees*	2,738	2.206	2.083	0	
*Self-employed*	1,187	2.366	2.139	−0.160	0.029
*Unemployed*	436	2.576	2.249	−0.370	0.001
**Stay later**					
*No*	2,658	2.170	2.126	0	
*Yes*	1,747	2.460	2.093	−0.289	<0.001
**No exercise**					
*No*	2,971	2.184	2.109	0	
*Yes*	1,434	2.494	2.121	−0.310	<0.001
**EWP**					
*0*	355	2.668	2.327	0	
*1*	862	2.173	2.054	0.490	<0.001
*2*	1,467	2.165	2.033	0.503	<0.001
*3*	1,672	2.366	2.158	0.291	0.018

As to the *living alone* population, the number of participants living alone is 1,582, which takes up a proportion of 35.91% of all samples, which indicates that living alone is a common phenomenon in these developed regions of China. Furthermore, the mean of the participants who lived alone was 2.550, and that of the population who lived with others was 2.137, and the *p*-value is smaller than 0.001; the mean difference is significant. This indicates that the people living alone had higher average GHQ−12 mental health scores (see rows 4–5 in Table 1). Regarding the GHQ-12 descriptive results of other control variables, the mental health score differed for different populations. Specifically, males get a higher mean GHQ-12 score, meaning men are more prone to have mental health problems during the COVID-19 pandemic than women. The younger population gets a higher GHQ-12 score. The GHQ-12 mean scores of the rural *and urban hukou residents were not much different, and the P-value is 0.416, which* is not notable. The participants with higher education levels get higher GHQ-12 scores than those with lower education. People with a lover get lower GHQ-12 scores. However, the mean difference of Senior high school compared to J.H. or Below is insignificant, and the *P*-value is 0.541. Population with more kids gets lower GHQ12 scores. Employees get lower GHQ-12 scores for the employment status factor. Individuals who stayed later get a higher mean GHQ-12 score for lifestyle factors. Individuals who did exercise regularly get a lower GHQ-12 score. The population that didn't use We-Media platforms gets the highest GHQ-12 scores.

### Multi-level regression analyses

Three models were performed in the study. The explanatory variable, living alone, and the explained variable, mental health score, were included in Model 1. Personal information about the social-demographic factors and lifestyle factors are added in Model 2 and Model 3, respectively. The regression results are given in Table 2. Model 1 shows a significant correlation between living alone and GHQ-12 score. The coefficient is 0.413 (*p* < 0.01). The significant correlation is also kept in Model 2 and Model 3. The corresponding coefficients are 0.172 (*p* < 0.01) and 0.151 (*p* < 0.05), respectively. This is consistent with hypothesis H1: living alone has a negative effect on the mental health of the participants.

**Table 2:** Regression results of effect of the *live alone* on mental health.

**Variables**	**Model 1**		**Model 2**		**Model 3**	
	**β (S.E.)**	**t**	**β (S.E.)**	**t**	**β (S.E.)**	**t**
**Live alone (no live alone** **=** **0)**	0.413***	6.240	0.172**	2.285	0.151**	2.018
	(0.066)		(0.075)		(0.075)	
**Gender (male** **=** **0)**			−0.225***	−3.537	−0.233***	−3.642
			(0.064)		(0.064)	
**Age**			−0.021***	−4.529	−0.019***	−4.177
			(0.005)		(0.005)	
**Hukou (Rural** ***Hukou*** **=** **0)**			0.035	0.525	0.057	0.865
			(0.066)		(0.066)	
**Education (** ***JH or Below** **=** **0*** **)**						
*Senior high school*			−0.212**	−2.287	−0.189**	−2.057
			(0.093)		(0.092)	
*C/U or above*			−0.135	−1.394	−0.098	−1.016
			(0.097)		(0.097)	
**With a lover (no** **=** **0)**			−0.026	−0.324	−0.033	−0.422
			(0.079)		(0.079)	
**Children (0 kid** **=** **0)**			−0.145***	−2.729	−0.133**	−2.515
			(0.053)		(0.053)	
**Employment (employees=** **0 )**						
*Self-employed*			0.243***	3.212	0.218***	2.895
			(0.076)		(0.075)	
*Unemployed*			0.473***	4.314	0.433***	3.971
			(0.110)		(0.109)	
**Stay later (no** **=** **0)**					−0.088	−1.180
					(0.074)	
**No exercise (do exercise** **=** **0)**					0.110	1.475
					(0.075)	
**Skipping meals (no** **=** **0)**					0.572***	6.849
					(0.084)	
**EWP**					−0.078**	−2.244
					(0.035)	
**Constant**	2.137***	53.849	3.113***	17.764	3.078***	15.628
	(0.040)		(0.175)		(0.197)	
**Observations**	4,405	4,405	4,405	4,405	4,405	4,405
**R-squared**	0.009	0.009	0.033	0.033	0.048	0.048

Significance level, *** p <0.01, ** p < 0.05.

Among other social-demographic variables, gender, age, education, number of children, and employment all have significant relationships with the GHQ-12 score in Model 2 and Model3. Among lifestyle variables, skipping meals, and entertainment on the internet (EWP) also have significant correlations to GHQ-12 score in Model 3.

Regarding personal information factors, first, the coefficient of the gender is −0.225 in Model 2 (*p* < 0.01) and −0.233 in Model 3 (*p* < 0.01), which presents that males are prone to have mental health problems during the COVID-19 pandemic. Second, the coefficients of the factor age are −0.021 (*p* < 0.01) in Model 2 and −0.019 (*p* < 0.01) in Model 3, indicating that the factor age has a negative correlation to mental health. Younger people are more likely to have mental health problems. Third, as to education, individuals who graduated from the senior high school is less likely to have mental health problems than those who graduated from J.H. or below. Fourth, for the factor of the number of kids, the coefficient is −0.145 (*p* < 0.01) in Model 2 and −0.133 (*p* < 0.01) in Model 3, which implies that more kids are positively related to mental health, indicating that people with more children can improve their mental health. Fifth, for the factor of employment, the employees are least likely to have mental health problems, then the self-employed population, and the unemployed population is most likely to have mental health problems during the COVID-19 pandemic.

Concerning the lifestyle, first, the coefficient of skipping meals is 0.572 (*p* < 0.01), indicating that individuals who used to skipping meals were more prone to have mental health problems. Second, EWP has a strong negative effect on the GHQ-12 scores, which means the anticipants who did not entertain on We-Media platforms are more prone to have mental health problems.

When it comes to R-squared, the values of the three models are 0.009, 0.033, and 0.048, respectively, which doesn't mean it's bad, unworthy of being interpreted, or useless. The point of our study is the effect of living alone on people's mental health. The model used living arrangements as an indicator of living alone and included a number of personal and demographic control variables. However, it cannot encompass everything that might affect a person's mental health. Considering all affecting factors, living alone is certainly not a major factor. The key was to see if there were small, reliable relationships, and there were. This small effect size also makes scientific sense. The R-squared values in Tables 3–**7** are small and can be interpreted in a similar way.

**Table 3:** Effect of the living alone on mental health, by gender.

**Variables**	**Men**		**Women**	
	**β (S.E.)**	**t**	**β (S.E.)**	**t**
Living alone	0.326^***^	3.408	0.484^***^	5.268
	(0.096)			
Constant	2.260^***^	38.191	2.027^***^	38.096
	(0.059)		(0.053)	
Observations	2,158		2,247	
R-squared	0.005		0.012	

Significance level, *** p <0.01.

### Heterogeneity in different groups

First, all participants are grouped into two parts: males and females. The target is to examine the heterogeneity effect of living alone on mental health between males and females. The regression results are listed in Table 3. Living alone is strongly correlated to the mental health of both males and females. It is interesting to note that males are more likely to have mental health problems during the pandemic. However, here living alone coefficient for men is 0.326 (*p* < 0.01), and that for women is 0.484 (*p* < 0.01), indicating that living alone has a stronger adverse effect on the mental health of the females, which supports hypothesis H2. The reasons may be that females are more sensitive and emotional. During COVID-19 pandemic, people living alone would face more difficulties for several changes caused by COVID-19.

Then, the participants are divided into three groups by age; population aged 16–35, 36–45, and 46–70. The results are given in Table 4. There is a significant correlation between living alone and the mental health of the younger population aged 16–25, with a coefficient 0.352 (*p* < 0.01). This indicates that the younger population living alone is more prone to have mental health problems. The significant relationship also exists in the population aged 46–47. However, the interesting result is that the coefficient is −0.606 (*p* < 0.01), which implies that living alone instead can improve the mental health of the older participants (aged 46–70). For the population aged 16–35, living alone has no notable effect on their mental health. It is not consistent with our prediction in H3.

**Table 4:** Effect of the live alone on mental health, by age.

**Variables**	**16–35**		**36–45**		**46–70**	
	**β (S.E.)**	**t**	**β (S.E.)**	**t**	**β (S.E.)**	**t**
Living alone	0.352^***^	4.551	0.264	1.053	−0.606^***^	−2.606
	(0.077)		(0.250)		(0.233)	
Constant	2.286^***^	43.397	2.010^***^	25.807	1.809^***^	20.888
	(0.053)		(0.078)		(0.087)	
Observations	3,081		754		570	
R-squared	0.007		0.001		0.012	

Significance level, *** p <0.01.

Table 5 gives the regression results of the effect of living alone on mental health by different employment status groups. All samples are divided into three groups: employees, self-employed, and unemployed (see Table 5). Contrary to our prediction in H4, living alone is significantly related to the mental health of employees (*p* < 0.01) and self-employed people (*p* < 0.01). However, this significant relationship does not exist in the unemployed population. The coefficients of living alone in the employee group and self-employed group are 0.374 and 0.723, respectively. This implies that living alone has a stronger adverse effect on the mental health of self-employed people than that of employees. We expect that unemployed persons who live alone may be under more stress during the COVID-19 pandemic. However, the result showed that living alone has no significant effect on the mental health of unemployed persons. The reason may be that whether they live alone or not, the unemployed population faced more difficulties and has poorer mental health.

**Table 5:** Effect of live alone on mental health, by employment.

**Variables**	**Employees**		**Self-employed**		**Unemployed**	
	**β (S.E.)**	**t**	**β (S.E.)**	**t**	**β (S.E.)**	**t**
Living alone	0.374^***^	4.666	0.723^***^	5.403	0.203	0.817
	(0.080)		(0.134)		(0.248)	
Constant	2.055^***^	40.455	2.148^***^	29.273	2.525^***^	20.259
	(0.051)		(0.073)		(0.125)	
Observations	2,782		1,187		436	
R-squared	0.008		0.024		0.002	

Significance level, *** p <0.01.

The regression results of effects of living alone on mental health for two groups (populations with a lover and without a lover) are given in Table 6. Living alone has a strong correlation with mental health for those with a lover and without a lover (*p* < 0.01). The coefficients of them are 0.328 and 0.305, respectively, indicating that effects of living alone on mental health of the two population are similar, and those living alone without lover is a little more prone to have mental health problems. The result is consistent with hypothesis H5.

**Table 6:** Effect of the live alone on mental health, by *with a lover*.

**Variables**	**Without a lover**		**With a lover**	
	**β (S.E.)**	**t**	**β (S.E.)**	**t**
Living alone	0.328^***^	2.779	0.305^***^	3.429
	(0.118)		(0.089)	
Constant	2.352^***^	25.588	2.082^***^	47.956
	(0.092)		(0.043)	
Observations	1,446		2,959	
R-squared	0.005		0.004	

Significance level, *** p <0.01.

Table 7 gives the regression results of living alone effect on mental health by EWP. Living alone has a significant relationship with mental health for populations use none, use two and use three, but has no notable correlation with population use one. The coefficients of the populations use none, use two and use three are 0.526 (*p* < 0.1), 0.569 (*p* < 0.01), and 0.352 (*p* < 0.01), respectively. This illustrates that living alone's effect on mental health is strongest for people using two platforms, then the living alone people did not have entertainment on the three platforms, living alone had the least effect on mental health for the population using three platforms. For the population using one platform, living alone effect on mental health is not notable. The results are consistent with the prediction in H6, the right amount of online entertainment can attenuate the living alone effect on mental health, and help improve the mental health of the living alone economically activity population during the pandemic time, which supports the prediction of H6.

**Table 7:** Effect of the live alone on mental health, by EWP.

**Variables**	**Use none**		**Use one**		**Use two**		**Use three**	
	**β (SE)**	**t**	**β (SE)**	**t**	**β (SE)**	**t**	**β (SE)**	**t**
Living alone	0.526^*^	1.938	0.206	1.369	0.569^***^	5.083	0.352^***^	3.333
	(0.271)		(0.151)		(0.112)		(0.106)	
Constant	2.514^***^	17.385	2.109^***^	24.988	1.976^***^	31.082	2.220^***^	32.038
	(0.145)	(0.084)	(0.064)	(0.069)
Observations	359		871		1,479		1,696	
R-squared	0.010		0.002		0.017		0.007	

Significance level, *** p <0.01,

* *p <0.1*.

## Discussion

The link between living alone and mental health has been extensively studied. However, the studies on effects of living alone on mental health during the COVID-19 are limited. Evidence on the mental health implications of living alone and the moderating effects of other socio-demographic features in Chinese population is especially rare. More studies need to be done to explore effects of living alone on Chinese population's mental health, especially for EAFP.

Analyzing the cross-sectional data from the questionnaires targeting to investigate the living alone results on the mental health of the EAFP in several developed regions of China, we found complex health implications of living alone among the EAFP in developed regions of China during the COVID 19 time. Living alone has a strong negative effect on the mental health of the EAFP. This finding lends some support to several previous research (9–11, 13, 23) that people who lived alone are more likely to have mental health problems.

The effect of living alone on mental health is more significant for females than males. Based on previous evidence on the health implications of living alone, we hypothesized that living alone's effect on mental health is more significant for women than men. The result is consistent with our hypothesis. For all samples, males are more prone to have mental health problems, this may be because men should be the breadwinners by tradition in China (31). The pandemic of COVID-19 may change their employment status, and influence their incomes, which results in their poorer mental health caused by continuous pressure. However, for living alone people, women are more sensitive and emotional. A study inducted in a university reports that women tend to have higher level of stress and other negative moods than men do (32). Besides, women may face more difficulties during the pandemic time, so they are more likely to be affected by living alone and result in mental health problems.

Whether living alone is a positive or negative factor for mental health depends on age. Living alone is stronger correlated with mental health of seniors (aged 46–70) and younger people (aged 16–35). The interesting result is that, the effect of living alone on the mental health of younger people (aged 16–35) is negative, indicating living alone lowers their mental health. However, this effect is positive for older people (aged 46–70), indicating that living alone can improve their mental health. For middle-aged people (aged 36–45), the living alone effect on mental health is not notable. To investigate the reasons, we thought from the following aspects. First, for younger people, some of them have just entered society, the economic situation is not well, the employment is not so stable, and without mental maturity, living alone makes them more prone to be loneliness causing health problems, which also echoes some previously published works (11, 13, 23). For the older population aged 46–70, living alone has a more positive strong effect on their mental health. The result is consistent with the results of (33). For these persons, they are with mature mind and stable financial resources, generally in higher positions, so living alone makes them more relaxed and free, thus improves their mental health.

Other than the moderating effects of gender and age, employment status is also an influencing factor for living alone effect on mental health. Through the study, we have found that living alone has a significant correlation with employees and self-employed population, but with no significant relationship with that of the unemployed population. The self-employment population is most likely influenced by living alone on their mental health. For employees, kinds of difficulties may appear because of COVID-19, such as income reduction, increased working time, risk of losing jobs, socializing manner changed, risk of loneliness, causing their poorer mental health compared with people who live with others (34).

Compared to employees, self-employed population who lived alone faced more difficulties such as work management, pressure, more proprietary costs to have their self-employed work normally, which may cause their poorer mental health at greater risk. Interestingly, for unemployed population seeking for a job, the results are contrary to our prediction in H4. Living alone has no notable effect on their mental health. Considering this, the reason may be that whether living alone or not, they are most likely to be effected by the COVID-19 with pressure of future's life.

We have also examined with a lover as potential moderators. We found that living alone has a stronger correlation with mental health for both populations with a lover and without. Population living alone with a lover gets better mental health compared to those without. Consisted with some previous research (34, 35), living alone is positively associated with mental health for the population with a partner. A lover is a soul partner of a person, who can give them spiritual support and strength, makes them relaxed from pressure, and less likely to feel lonely, and accordingly improve their health. For factor of EWP, living alone has a strong significant effect on mental health for populations using none, two and three, but has no notable relationship with the population using one. This is consistent with our prediction in H6. No or frequent online entertainment would make the stronger effect of living alone on mental health. Considering the reasons, people can obtain some positive life information on the We-media platforms, which not only reduces their risk of loneliness, but also helps them build better living lifestyles, improves their living confidence, accordingly reduces the impacts of living alone on their mental health (36, 37). If they use We-media platforms too often, time spent on sleep and exercise may be taken up, worsening the effect of living alone on their mental health.

Therefore, the effect of living alone on the mental health of the EAFP during COVID 19 has generated new inequalities among different groups. The study provides new evidence to develop measures to improve the mental health of the EAEP in China. The government should provide more psychological support to EAFP living alone, and also should lead them to establish correct marriage view and love to reduce negative effect of living alone on their mental health.

The empirical study also has limitations. First, we have tried to select economically active floating population through diversity in the sampling process to make it more representative. However, there are some unemployed people for a too long time, thus making the samples not fully representative. However, in a study on migrant populations' mental health during the pandemic, the presence of these special samples had less impact on the effects of living alone on EAEP, and the results provide a valid argument and valuable insight into the mental health of economically active populations living alone. Another limitation of this study is that we cannot track the psychological changes caused by the change of residence status of individuals because we only use a cross-sectional data for the purpose that the impact of living alone on mental health can be more accurately assessed. In future work, we will track the impact of an individual's living arrangements over time on his mental health and assess its significance more accurately.

## Conclusion

The effect of living alone on mental health of the EAFP in several developed regions of China during the COVID-19 pandemic is explored in this study. The results show that living alone has a strong negative effect on mental health. The negative effect of living alone on mental health is stronger for females than males. Living alone has a significantly negative effect on the mental health of population aged 16–35 while it has a significantly positive effect on the mental health of those aged 46–70, and has no notable effect on those aged 36–45. The significant effects of living alone on mental health were stronger for self-employment population than that for employees, and not notable for unemployed ones. The effects of living alone on mental health were stronger for people with a lover than those without. Never or sometimes entertaining online both enhance the effects of living alone on the mental health for EAFP. Therefore, aside from providing more public services to give more support to struggle against the pandemic, the government should give more psychological support to those living alone with guidance to a correct view of marriage and love and moderate use of online entertainments to reduce the negative effects of living alone and prevent them from mental health problems.

## Data availability statement

The raw data supporting the conclusions of this article will be made available by the authors, without undue reservation.

## Ethics statement

This study was approved by the Research Ethics Committee of South China Normal University. Informed consent from all participants was obtained at the start of the questionnaire, and all participants voluntarily participated and were free to withdraw from the questionnaire at any time.

## Author contributions

JX and LZ contributed to conception, data interpretation, revised, and fixed the draft. JX made statistical analysis. LZ completed the first draft. Both authors contributed to manuscript revision, proofread, and approved the submitted version.

## Funding

This study was supported by the scientific research project of Education Department of Hubei Province (No. B2021250).

## Conflict of interest

The authors declare that the research was conducted in the absence of any commercial or financial relationships that could be construed as a potential conflict of interest.

## Publisher's note

All claims expressed in this article are solely those of the authors and do not necessarily represent those of their affiliated organizations, or those of the publisher, the editors and the reviewers. Any product that may be evaluated in this article, or claim that may be made by its manufacturer, is not guaranteed or endorsed by the publisher.

## Footnotes

^1^ http://www.stats.gov.cn/tjsj/zxfb/202007/t20200717_1776516.html

^2^Douyin, Xinlang micro-blog, and WeChat are three popular We Media platforms in China.
